# Costs of implementing and sustaining enhanced collaborative care programs involving community partners

**DOI:** 10.1186/s13012-019-0882-6

**Published:** 2019-04-18

**Authors:** Theresa J. Hoeft, Heather Wilcox, Ladson Hinton, Jürgen Unützer

**Affiliations:** 1 Department of Psychiatry and Behavioral Sciences, University of Washington School of Medicine, Box 356560, Seattle, WA 98195-6560 USA; 2 Department of Psychiatry and Behavioral Sciences, University of California, Davis, CA USA

**Keywords:** Collaborative care implementation, Adaptation, Community partners, Family, Depression, Older adults

## Abstract

**Background:**

Collaborative care is an evidence-based program for treating depression in primary care. We sought to expand this model by recruiting clinics interested in incorporating community partners (i.e., community-based organizations (CBO) and/or family members) in the care team. Seven sites implemented evidence-based collaborative care programs with community partners while collecting information on costs of implementing and sustaining programs.

**Methods:**

Sites retrospectively collected data on planning and implementation costs with technical assistance from study researchers. Sites also prospectively collected cost of care activities over a 1-month period once the program was implemented to determine resources needed to sustain programs. Personnel salary costs were adjusted, adding 30% for benefits and 30% for administrative overhead.

**Results:**

The programs implemented varied considerably in staffing, involvement of care partners, and allocation of costs. Total planning and implementation costs varied from $39,280 to $60,575. The largest implementation cost category involved workflow development and ranged from $16,325 to $31,375 with the highest costs in this category attributed to the most successful implementation among clinic-CBO programs. Following implementation, cost per patient over the 1-month period ranged from $154 to $544. Ongoing strategic decision-making and administrative costs, which were included in cost of care, ranged from $284 to $2328 for the month.

**Conclusions:**

Sites implemented collaborative care through differing partnerships, staffing, and related costs. Costs to implement and sustain programs developed in partnership are often not collected but are crucial to understanding financial aspects of developing sustainable partnerships. Assessing such costs is feasible and can inform future partnership efforts.

**Electronic supplementary material:**

The online version of this article (10.1186/s13012-019-0882-6) contains supplementary material, which is available to authorized users.

## Background

Depression is common in older adults and comes at a high cost to patients and their families. Major depression affects 2–5% of community-dwelling older adults and 5–10% of older adults in primary care settings [1–4]. Late-life depression impairs quality of life [5] and ability to function and enjoy old age [6]. It is associated with increased healthcare costs [7, 8], family stress, and increased risk of suicide. Depression is the most important—and most treatable—risk factor for suicide [9].

Over the past two decades, significant progress has been made in our ability to diagnose and treat depression in older adults. Research has demonstrated that collaborative care programs in which primary care providers (PCPs) are supported by mental health professionals to treat depression in older adults can dramatically improve the effectiveness and cost-effectiveness of depression treatment [10–13]. Collaborative care involves a primary care-based team, including a care manager who tracks depression symptoms and offers brief psychotherapy and/or medication management, a psychiatric consultant who consults with the care manager on a weekly basis about a caseload of patients tracked in a registry, and the patient’s PCP.

Despite these advances, many depressed older adults either do not access treatment or fail to engage sufficiently in treatment to benefit. Vulnerable groups at particularly high risk for ineffective depression care include minorities, older men, and older adults with multiple medical problems, less formal education, or lower socioeconomic status [14–16]. Closing gaps in care to improve access to effective depression treatment is important and timely.

One of the most promising approaches to improving the reach and effectiveness of late-life depression care builds on task shifting, or task sharing, in mental health care delivery [17] which involves sharing care delivery tasks with typically less highly skilled clinicians and staff to allow each to work to the top of their license [18–20]. Task sharing can enhance care by broadening the scope of care delivery tasks. Collaborative care, for example, includes repeated depression symptom monitoring and tracking patients in a registry which were previously not standard elements of depression care. A review of the literature on future directions for late-life depression care identified the promise of task sharing to involve family members and community-based organizations (CBO) in depression care [16]. Often, these community partners have eyes and ears in the community and thus can enhance depression care through improving access to care, engagement in treatment both initially and during depression relapse, and the patient care experience for depressed older adults.

As part of a late-life depression initiative in California, seven sites were awarded funding for a period of 2 years to implement an enhanced collaborative care model that incorporated a CBO and/or family member(s) of the older adult in the collaborative care team. Core principles of collaborative care were maintained in the implementation, so modifications only involved expanding the care team and offering care in settings outside primary care (e.g., CBOs and home visits). A task matrix of all collaborative care tasks ensured programs included all aspects of collaborative care.

Costs associated with program implementation and operation are infrequently collected but critical to program success and sustainability. As sites implemented their partnered models of care in 2015, a program evaluation included assessment of (1) planning and implementation costs and (2) costs to operate and sustain the model of care following the implementation period. Costs were assessed based on detailed spreadsheets of tasks completed and associated hours, salaries, and non-personnel costs. This information enabled us to study ways in which tasks are shared across organizations; the overall costs associated with planning, implementation, and cost of care delivery; and additional costs that may be attributed to working in partnership.

As evidenced within recent developments in the USA of Accountable Care Organizations and Accountable Health Communities, value-driven health care is increasingly looking to community partnerships to improve care for complex patients [21, 22]. Costs associated with working in partnership are not typically studied but are crucial to understanding resources necessary to carry out this important work. This study outlines costs associated with partnered implementation of collaborative care, a process however that could be applied to any implementation of an evidence-based practice.

## Methods

### Data collection

Two pilot sites gave feedback on the data collection plan, guidance documents, and preferences for reporting results back to the sites. One person at each organization who was most familiar with project activities utilized Excel® spreadsheets to collect project-specific activities (i.e., activity description, time spent, salaries, non-personnel costs) for both the planning + implementation periods and the 1-month care delivery period which fell within the sustainment period. Spreadsheet items are further described in Additional file 1. Planning and implementation periods were delineated by the notice of grant award, enrollment of the first patient, and date when the site’s major workflow changes came to an end. The decision to end the implementation period when workflow changes declined was based on feedback from the pilot sites that a standard 3- or 6-month period for the implementation timeframe would not fit each site’s implementation. The resulting implementation periods ranged from 1.5 months (through January 2016) to almost 9 months (through August 2016). The sustainment period, when cost of care data was collected, followed the implementation period. Most sites completed the cost of care spreadsheet in October 2019 which fell between 2 and 9 months after their implementation period ended and sustainment period began.

Study researchers supported data collection at each site through a minimum of two telephone calls, reviewing drafts of spreadsheets to anticipate issues and questions, and responding to additional questions by email and telephone. Since the cost evaluation study began 6 months after the sites’ grants were awarded, sites retrospectively collected costs associated with planning + implementation activities. Once the model was implemented, sites prospectively recorded 1-month cost of care activities. Midway through the cost of care data collection, sites were asked to submit a draft spreadsheet to address questions and anticipate potential missing data early on.

Data collection spreadsheets separated costs into pre-specified categories of interest based largely on a previous study of collaborative care in the COMPASS implementation [23, 24]. Planning + implementation costs were collected within the categories of developing and training new roles, developing workflows and preparing existing staff, project oversight and strategic decision-making, registry/IT expenses, and other costs. Each organization then additionally separated activities on the spreadsheets by whether they occurred in the planning period or implementation period. Costs of care activities were collected under major categories associated with collaborative care including care manager time, psychiatric consultant + PCP champion time, strategic decision-making, care manager supervision + administrative management, registry/IT and quality improvement (QI), and other costs.

### Analysis

While the pre-specified cost categories in the spreadsheets informed the cost summaries, review of the spreadsheets suggested additional categories. The majority of activities reported in this partnered implementation involved workflow development and other planning costs specifically done either in partnership or individually within each organization. Details of care managers’ time revealed additional richness of the data. We thus used the original categories as a guide, but allowed for new areas of synthesis to be revealed in the data—an approach similar to deductive and inductive qualitative research. Each activity had an associated number of hours rounded to the quarter hour and associated salary for all staff members in the activity. Sites were given an option to provide actual salaries or average salaries for the position. For one site without salary information, we used the Bureau of Labor Statistics salary information for that region in California. Costs were summarized by a priori categories of interest and categories later identified upon review of the cost data. Personnel salary costs were adjusted by adding 30% for benefits and an additional 30% for administrative overhead. Patient demographic differences across sites in Table 1 were assessed using Pearson’s chi-square test.

**Table 1:** Patient participant demographics by site

	Site 1 (*n* = 51)	Site 2 (*n* = 11)	Site 3 (*n* = 24)	Site 4 (*n* = 47)	Site 5 (*n* = 75)	Site 6 (*n* = 37)	Site 7 (*n* = 29)	Total (*n* = 274)	*p* value
Age, *n* (%)									
65–69	28 (54.9)	5 (45.5)	13 (54.2)	3 (6.4)	29 (38.7)	21 (56.8)	7 (24.1)	106 (38.7)	
70–74	12 (23.5)	2 (18.2)	7 (29.2)	8 (17.0)	17 (22.7)	8 (21.6)	10 (34.5)	64 (23.4)	
75–79	5 (9.8)	2 (18.2)	2 (8.3)	9 (19.1)	13 (17.3)	5 (13.5)	6 (20.7)	42 (15.3)	
80+	6 (11.8)	2 (18.2)	2 (8.3)	27 (57.4)	16 (21.3)	3 (8.1)	6 (20.7)	62 (22.6)	< 0.001
Gender, *n* (%)									
Male	20 (39.2)	0 (0.0)	14 (58.3)	16 (34)	14 (18.7)	11 (29.7)	28 (96.6)	103 (37.6)	
Female	31 (60.8)	11 (100)	10 (41.7)	31 (66)	61 (81.3)	26 (70.3)	1 (3.4)	171 (62.4)	< 0.001
Race/ethnicity, *n* (%)									
Hispanic	20 (39.2)	7 (63.6)	1 (4.2)	6 (12.8)	26 (34.7)	37 (100)	5 (17.2)	102 (37.2)	
Black	9 (17.6)	1 (9.1)	10 (41.7)	3 (6.4)	0 (0.0)	0 (0.0)	1 (3.4)	24 (8.8)	
Asian	3 (5.9)	0 (0.0)	0 (0.0)	7 (14.9)	2 (2.7)	0 (0.0)	4 (13.8)	16 (5.8)	
Multiple reported	1 (2.0)	0 (0.0)	0 (0.0)	1 (2.1)	2 (2.7)	0 (0.0)	0 (0.0)	4 (1.5)	
White, non-Hispanic	16 (31.4)	2 (18.2)	11 (45.8)	26 (55.3)	44 (58.7)	0 (0.0)	19 (65.5)	118 (43.1)	
Missing	2 (3.9)	1 (9.1)	2 (8.3)	4 (8.5)	1 (1.3)	0 (0.0)	0 (0.0)	10 (3.6)	< 0.001
Interpreter needed, *n* (%)	1 (2.0)	5 (45.5)	0 (0.0)	0 (0.0)	0 (0.0)	1 (2.7)	0 (0.0)	7 (2.6)	< 0.001
Preferred language, *n* (%)									
English	35 (68.6)	4 (36.4)	24 (100.0)	47 (100.0)	54 (72.0)	0 (0.0)	29 (100.0)	193 (70.4)	
Spanish	14 (27.5)	6 (54.5)	0 (0.0)	0 (0.0)	21 (28.0)	37 (100)	0 (0.0)	78 (25.8)	
Other	2 (4.0)	1 (9.1)	0 (0.0)	0 (0.0)	0 (0.0)	0 (0.0)	0 (0.0)	3 (1.2)	< 0.001

## Results

### Sites overview

Sites selected to join the initiative all tended to work with low-income, diverse populations as is described through program participants in Table 1. By design, all patients were 65+ years old. Two sites saw a substantial number of patients across all age groups while four sites recruited primarily older adults who were 65–69 years of age. One site working with primarily older home-bound individuals enrolled a majority of their patients in the 80+ age group (site 4). Two sites served predominantly men (96.6% in a VA clinic (site 7) and 58.3% in a non-VA clinic (site 3)), while a site utilizing female community health workers (CHWs) offering home visits served only women (100% of enrollees) (site 2). Two sites served a predominately Hispanic population (63.6% and 100%) (sites 2 and 6), while other sites varied in their level of engagement with diverse groups. Finally, the patients’ preferred language was often Spanish, particularly at the two sites that enrolled a higher percentage of Hispanic participants.

Sites differed in types of partnerships they developed alongside primary care and size of their primary care networks. Three sites involved a senior center as the CBO partner (sites 1, 3, and 6). Three other sites offered a majority of care via home visits delivered by CBO staff from a health education outreach organization (site 2), an in-home supportive services organization focusing on in-home psychotherapy (site 4), and a health and human services agency (site 5). Two sites involved family in care (sites 6 and 7), though site 7 did not include a CBO partner. Each site started their program development at one clinic with the exception of site 6 which started in two clinics. Most clinic partners fell within organizations comprised of three or more primary care clinics with the exception of sites 2 and 5.

**Fig. 1:**
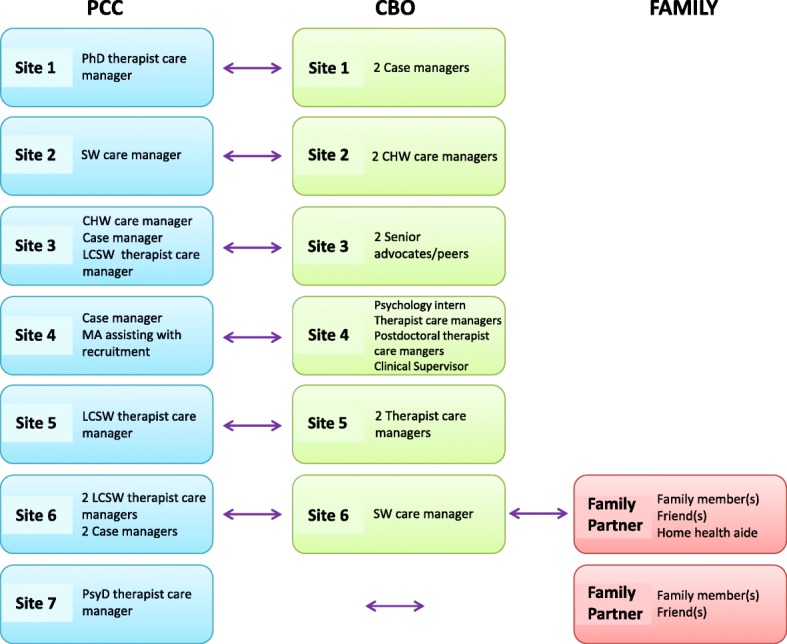
Care manager team composition variation across sites. PCC, primary care clinic; CBO, community-based organization; SW, social worker; LCSW, licensed clinical social worker; CHW, community health worker

Sites also differed considerably in terms of staffing composition (e.g., professional background and licensure of staff), with staffing composition sometimes changing mid-way through the grant period due to either turnover or development of a new clinical workflow. Further details on the original mix of care managers at each site are listed in Fig. 1. Sites 2 and 3 employed CHWs with varying levels of training. Sites 1 and 7 employed PsyD- or PhD-trained therapists, though following turnover in this position, site 1 shifted to utilizing licensed clinical social worker (LCSW) staff. Site 6 mixed master’s-level therapists with bachelor’s-level case managers in the clinic setting to split regular depression symptom monitoring and case management tasks. Site 6 also paired the clinic team with a bachelor’s-level social worker from the senior center to enhance family engagement in care and facilitate referrals between the two organizations. Site 4 utilized psychology interns, their supervisor, and a postdoctoral fellow for additional support to offer psychotherapy in the home and monitor symptoms. One site that originally relied on two master’s-level case managers at the senior center to help engage patients in care ended their formal partnership with the senior center for this project mid-grant (site 1). The clinic then hired a case manager to join the clinic care team to more proactively reach out to older adults who may need local social services, connect them to these services, and report back to care managers and the psychiatric consultant on the patients’ progress in reaching needed services.

Care processes also differed across sites and consequently influenced costs of care. For example, collaborative care typically involves a blend of office-based clinic visits and phone contacts to improve engagement in care and opportunities for the care managers to check in on patients. Home visits were a unique aspect of care across several sites in this implementation project. In-person visits with a care manager at the clinic ranged from less than one visit (0.62 visit) to 18.9 visits on average per person. Clinic visits should have been one or greater per patient to indicate sharing of depression care and clinic involvement (e.g., to help rule out physical causes of depression). Sites on the lower end of this range for clinic-based care tended to be CBOs with weaker connections to primary care (sites 2 and 4), while the site with the most in-person visits employed a large team of care managers at the clinics who reached out on a regular basis. Most sites, however, provided on average 3.0 to 7.8 clinic visits per patient. Two of the three programs that included home visits in care involved fairly minimal care management activities within the clinic system (sites 2 and 4), while the third located the home-visiting care manager in a shared office at the clinic with the LCSW clinic care manager and actively shared care on their caseload of patients (site 5). Phone contact was low across sites with the exception of two sites which provided 5.0 and 8.2 calls per patient on average (sites 5 and 6 respectively). Site 6 thus had the highest number of phone and clinic visits per patient. Three sites offered home visits, and patients received on average 3.3, 8.4, and 9.8 home visits (sites 5, 2, and 4 respectively). Site 4 thus had the lowest number of phone and clinic visits and the highest number of home visits. Care was offered in-person at the CBO, but this was infrequent.

Involvement of the psychiatric consultant in care is another aspect that influenced costs. At six of the sites, the total percent of patients enrolled that were ever discussed at a weekly case review with the psychiatric consultant ranged from 39.2 to 65.3%. The seventh site discussed 100% of their patients with the psychiatric consultant, though the site enrolled few patients overall perhaps making these cases easier to discuss (site 2).

In terms of depression treatment outcomes, once engaged in the program, all sites were able to improve depression symptoms for patients as measured by the Patient Health Questionnaire-9 (PHQ-9). Of the 274 patients enrolled in care, 247 patients had two or more visits when a PHQ-9 was recorded. For this sample, 62% of patients showed a 50% or greater improvement from their initial PHQ-9 score and/or their last PHQ-9 score was less than 10, while 59% showed improvement of 5 points or more from their initial PHQ-9 score. A total of 32% achieved a score of less than 5 on their final PHQ-9, which is considered to be remission from depression.

### Planning + implementation costs

Complete planning and implementation cost data were collected on four sites, three involving a CBO and one including family care partners. Site 6 declined to participate due to high turnover in key leadership and administrative positions and thus lost critical knowledge about planning and implementation activities. Two sites (sites 1 and 2) offered incomplete data due to the ending of their formal partnerships near the due date for data collection, though several of their costs can be compared to other sites (e.g., clinic costs spent on workflow development). The clinic contact at site 2 omitted the clinic implementation costs. The time the clinic spent on joint activities with the CBO, however, were estimated from the CBO’s documentation of clinic meeting attendees at joint administrative meetings in the implementation period. The CBO contact at site 1 could not be reached to collect planning and implementation cost data after their formal partnership for the program ended.

**Fig. 2:**
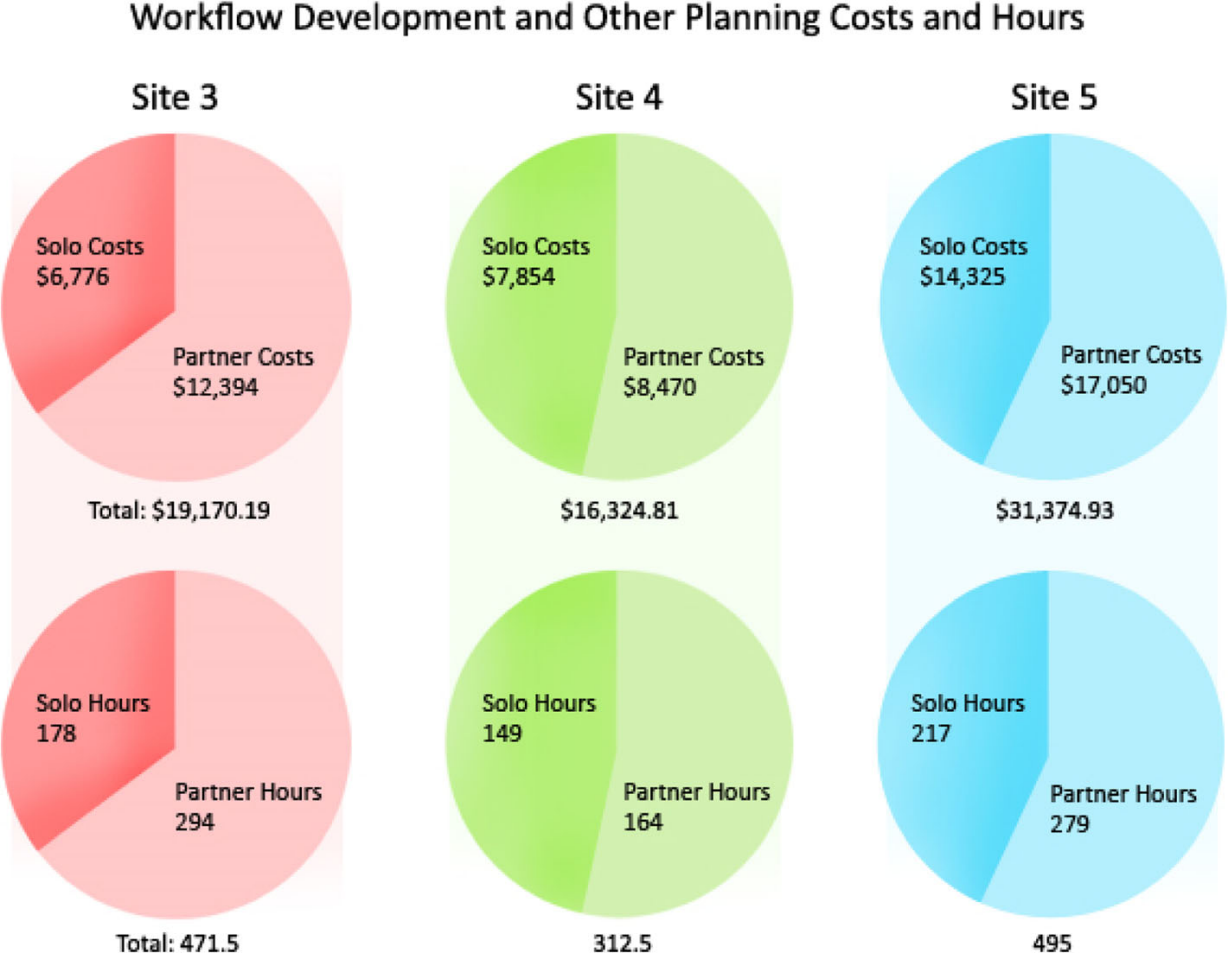
Total workflow development and other planning costs spent independently and in partnership. Totals for costs and hours indicate totals for workflow development and other planning costs only

Planning and implementation costs by type of expense (e.g., training, workflow development, other planning activities) are presented in Table 2. For sites involving a CBO partner, the majority (52–65%) of costs were associated with shared workflow development and non-workflow-related planning (Fig. 2). The most successful clinic-CBO implementation (site 5) accrued the highest costs for workflow and non-workflow planning activities, totaling $31,375 (Table 2). In contrast, total costs for workflow development and other planning activities at the other clinic-CBO sites with complete data were $16,325 and $19,170 (Fig. 2). At the family care partner clinic that did not partner with a CBO (site 7), these workflow and non-workflow planning activities totaled $16,809.

**Table 2:** Planning and implementation costs across sites by organization and expense category (cost and time spent)

Expense category	Site 3 clinic^a^	CBO partner	Site 4 CBO^a^	Clinic partner	Site 5 CBO^a^	Clinic partner	Family site 7 clinic^a^	Site 1 clinic^ab^	Site 2 CBO^b^
Training: new roles ($, hours)	$2958.67	$1030.90	$8327.10	$898.17	$1359.07	$2487.55	$1718.86	$12,015.25	$9822.80
	138.5	26.5	262	15	27	44.5	55	112.5	491
Training: existing staff in existing roles ($, hours)	$327.60	$995.80	$1211.29	$4594.49	$2334.04	$2273.34	$6284.43	$1788.15	$773.50
	9	26	19	37	38	23	75.5	29.5	35
Total for training ($, hours)	$6872.97		$15,031.04		$8454.00		$8003.29	$13,803.40	$10,596.30
	200		333		132.5		130.5	142	526
Workflow development: with partner ($, hours)	$2204.80	$2204.80	$1462.45	$2330.69	$3881.81	$5508.89		$2850.90	$665.60
	58	56	50	33	68.5	86		45	29
Workflow development: solo planning ($, hours)	$1410.50	$325.65	$1241.86	$640.64	$3370.29	$1457.64	$4646.86	$470.60	$6319.30
	43.5	8.5	36.5	5	57	13	56	8	283.5
Total for workflow development ($, hours)	$6145.75		$5675.64		$14,218.63		$4646.86	$3321.50	$6984.90
	166		124.5		224.5		56	53	312.5
Other planning activities: with partner ($, hours)	$6205.94	$1778.40	$845.54	$3831.67	$4490.45	$3168.58		$2077.40	$2611.70
	135	45	44	36.5	73	51		30	112
Other planning activities: solo planning ($, hours)	$3976.70	$1063.40	$4178.17	$1793.79	$7552.21	$1945.06	$12,162.25	$1673.10	$4882.80
	104.5	21	93.5	14	117	29.5	125	28	197
Total for other planning activities ($, hours)	$13,024.44		$10,649.18		$17,156.30		$12,162.25	$3750.50	$7494.50
	305.5		188		270.5		125	58	309
Other costs ($)	$4171.84		$12,346.95		$6767.12		$9312.40	$2251.17	$7113.60

^a^Organization receiving grant

^b^Site 1 data is incomplete as CBO partner did not collect data. Site 2 had limited data from clinic’s implementation period

Training and other expense categories also varied by site. Training costs were higher for three sites (sites 1, 2 and 4). Site 4 is a training program for psychology interns, so many training activities for interns were offered by supervisors at the CBO, though notably, intern costs were documented as in-kind hours. Site 2 faced low patient enrollment and trouble recruiting and thus engaged staff in additional training activities (e.g., re-watching webinars) with available time not spent on patient care in the implementation period. Site 1 experienced turnover of two key leadership positions and their original care manager within the planning and implementation periods. Other costs varied in large part due to larger registry/IT costs at some sites (e.g., for the CBO to gain read/write or read-only access to the clinic’s electronic health record) or due to longer implementation periods that then covered the initiative’s annual meeting and thus related travel expenses.

Total planning and implementation costs for sites with complete data varied from $39,280 to $60,575 (Table 3). Three of the sites reported a majority of hours spent in the planning phase, while one site reported comparable hours in planning and implementation phases, in part due to a longer implementation period of 9 months. In clinic-CBO partnerships with complete data, the organization that received the grant spent more hours in the planning and implementation periods than their partner organization, though the difference in hours was more pronounced for sites 3 and 4 compared to site 5. Though CBO data was unavailable, clinic costs for site 1 were comparable to the other clinics’ costs in the planning and implementation periods. Implementation costs for the site 2 clinic are low due to limited data from this period that was reported by the CBO contact following turnover of the clinic contact.

**Table 3:** Total planning and implementation costs across sites by phases of implementation (cost and time spent)

Organization	Planning phase			Implementation phase			Total		
	Hours	Cost^b^	Days in phase	Hours	Cost^b^	Days in phase	Hours	Cost^b^	Cost + 30% overhead
Site 3 clinic^a^	256.5	$11,286.95		233	$10,136.42		489.5	$21,423.37	
CBO partner	100	$5462.33	155	83	$3329.30	269	183	$8791.63	$39,279.50
Site 4 CBO^a^	510.5	$25,339.65		27.5	$681.80		538	$26,021.45	
Clinic partner	158	$17,236.66	161	5.5	$444.70	72	163.5	$17,681.36	$56,813.65
Site 5 CBO^a^	301	$20,863.00		92.5	$5420.81		393.5	$26,283.81	
Clinic partner	221	$17,254.52	128	46	$3057.71	108	267	$20,312.23	$60,574.85
Family site 7 clinic	280.5	$29,402.93	248	61	$4721.89	55	341.5	$34,124.81	$44,362.25
Site 1 clinic^ac^	189	$16,179.37	182	73	$6947.20	163	262	$23,126.57	$30,064.54^c^
Site 2 CBO^a^	1012.5	$28,875.60		139	$3313.70		1151.5	$32,189.30	
Clinic partner^c^	141.5	$7416.26	252	15	$815.37	134	156.5	$8231.63	$52,547.21^c^

^a^Organization receiving grant

^b^Hourly wage + 30% benefits

^c^Site has incomplete data. Site 1 data is incomplete as CBO partner did not collect data. Site 2 had limited data from clinic’s implementation period, thus clinic implementation costs and total costs for site are underestimated

### Costs of care

We received complete cost of care data from four sites, three involving a CBO and one including family care partners. One CBO collected data even though their partner clinic declined participation after the clinic decided not to sustain the existing model (site 6). The CBO costs for this site amounted to $46 per patient treated that month, which are not reported in Table 4. One clinic collected data on costs after their partnership with the CBO formally ended, and they had implemented a new program that incorporated a new clinic case manager to actively engage patients in social services out in the community (site 1). Another site that saw their formal partnership dissolve was not able to collect cost of care data, as they did not implement their new model of care by the end of the grant period (site 2). Both partnerships ended in part due to an unexpectedly low number of patients that were using services at both partnering organizations (i.e., CBO and clinic).

**Table 4:**
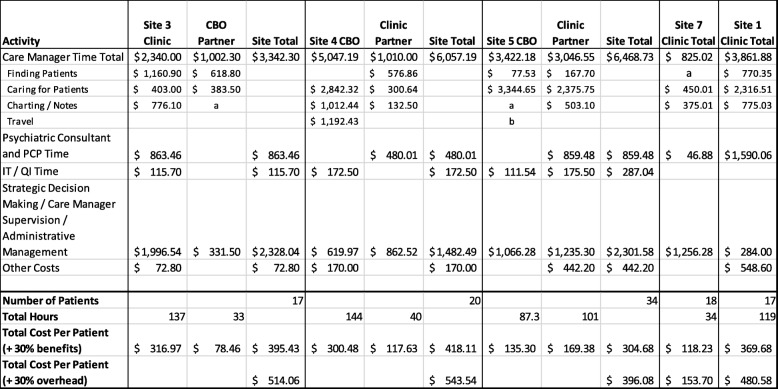
One month costs of care delivery in the sustainment period following implementation, total, and by number of patients treated during month

*a* costs included in time caring for patients category

*b* travel costs notably missing for this site that offered regular home visits

Total program costs per patient treated for 1 month of care, including the 30% overhead adjustment, ranged from $154 to $541 across sites with complete data. Across all sites, enrollment was relatively low, likely increasing the average cost per patient treated.

Care management costs varied by organization, and some organizations focused more on activities such as finding patients or caring for patients. Across clinic-CBO sites with complete data, one showed higher care manager costs for the CBO, one showed higher costs for the clinic, and the third had relatively equal costs between CBO and clinic care managers. Subcategories in the care manager activities were not delineated in the data collection spreadsheets but developed upon thematic review of these activities. Travel was an area of data collection that was not specifically requested as a care manager activity and was thus not consistently reported. Travel time is not typically part of collaborative care programs and was only reported for one site offering home visits (site 4). Total care manager costs across sites with complete data ranged from $825 to $3047 in clinics and $465 to $5047 in CBOs. The highest costs for care management among the CBOs did not account for 51 h of in-kind time from the intern care manager (site 4). Charting activities and time finding patients could not be consistently separated from caring for patients at all sites but are reported separately for sites that reported a clear delineation between these activities.

Costs related to strategic decision-making and program administration continued in this sustaining phase of implementation, as sites used meeting times to discuss patients and to continue to make changes to their programs. These costs ranged from $284 to $2328 total over the 1-month data collection period among the sites with complete data. These costs were particularly high for two of the clinic-CBO partnerships that typically involved a greater number of individuals in their administrative meetings (sites 3 and 5). Care manager supervision outside the weekly case review with the psychiatric consultant and administrative management were included in this category, as these activities often overlapped in regular meetings.

Other notable costs also varied across sites. Non-billable time for the psychiatric consultant and PCP champion ranged from $47 to $1590 total over the 1-month data collection period, though most sites reported between $480 and $859 on such costs. The site that utilized only $47 for psychiatric consultant involvement in care underutilized this resource both due to shorter weekly case reviews for half of the 1-month period and vacation falling over the other half of the month. IT and QI activities were minimal for most sites and typically involved a QI review of the caseloads to prepare progress reports to the grant funder. Such QI activities overlap with how sites are predicted to maintain oversight on care manager activities and plan for future patient workflow improvements; they are therefore included in Table 4 despite being considered grant-specific activities during the data collection period. Other staff and non-personnel costs were reported for three organizations but were minimal, with costs including front desk support for depression screening and travel expenses (e.g., mileage).

## Discussion

High costs of care delivery over 1 month can be largely attributed to low enrollment at all sites and continued costs of strategic decision-making/administrative support/supervision at all but site 1. The primary reasons for low enrollment were (1) the limited number of shared patients across the CBO and clinic for some sites and (2) low motivation among those with late-life depression to access care from the clinic and CBO if the CBO staff were not providing home visits. For example, older adults with depression from the clinic were reluctant to venture to a senior center even with additional supportive outreach and, for some, arranged transportation to the CBO. Site 1 had higher costs of psychiatric consultant and primary care champion time however which may have included some of these administrative activities such as advertising the program across the clinics and attempting to increase enrollment. Site 7 had notably low costs for psychiatric consultant/PCP time. Sites 4 and 5 had higher care manager costs, and both sites involved home visits by the CBO staff. In comparison with previous collaborative care studies without community linkages, these costs are high relative to the original IMPACT collaborative care study [10, 13] and TEAMCare study which focused on comorbid depression with physical illness (e.g., diabetes and/or cardiovascular disease) [25, 26] but more comparable to a recent implementation of TEAMCare known as the COMPASS study from which these methods were based [24, 27] (personal communication with Michael Maciosek on June 14, 2018). The methods in this study were developed to account for additional non-billable and often hidden costs (e.g., program administration) that may not be estimated in other cost of care studies. Continued strategic decision-making and administrative costs during the 1-month period were also higher than anticipated, perhaps due to sites’ ongoing efforts to increase enrollment in these new innovative programs involving family and/or CBO care partners. We plan to assess the cost of care again in the next year as sites continue to develop more scalable and sustainable programs (e.g., through increased enrollment and different staffing compositions).

As mentioned in the results, sites 1 and 2 lost their partnering organizations during the grant period. Site 1 revised their program to incorporate a case manager in the depression care team who is more active in working with CBOs out in the community. Site 2 eventually shifted to a home visit collaborative care program out in the community that allows them to work with community members seeing providers at a variety of primary care clinics. We have used the cost evaluation data to coach site 1 on sustainability of their new program and will collect data from site 2 in the coming year to help them with sustaining their new program as well.

Planning and implementation costs also varied across sites and were lower across organizations than comparable costs in the COMPASS study implementation of collaborative care (personal communication with Michael Maciosek on June 14, 2018). COMPASS planning and implementation costs supported implementation at health systems consisting of approximately 10–50 clinics, while most Care Partners sites implemented their program at one clinic initially explaining higher costs in COMPASS. The Care Partners site that spent the most overall in the planning + implementation periods and also the most on workflow development + other planning activities was the most successful model to develop within the first 2 years of the late-life initiative (site 5). This site’s success was evident based on (1) their efforts to build the infrastructure to communicate about patients and improve workflows when needed, (2) total patient recruitment, and (3) improved depression symptoms. We speculate that this might be related to the strength of each organization’s infrastructure and commitment of these partners to the partnered collaborative care program. Both organizations also brought to the table two evidence-based programs to improve late-life depression; the clinic implemented collaborative care, and the CBO used an existing Healthy Ideas program to reach individuals in their homes and offer additional depression education, behavioral activation, and case management support. Their complex patient population often consisted of patients with which they were working independently prior to the program’s development, and now the CBO was able to share insights to the clinic on the home environment and additional case management support in the home or by phone from the CBO home visiting care manager. In contrast, site 3 reported approximately the same amount of time on planning and implementation activities, though the split between the CBO and the clinic was less even and their total costs associated with this time was lower. Through the larger evaluation of implementations across the seven sites within which this smaller cost study fell, we learned that site 3 struggled to include higher-level leadership as champions for improving depression screening, depression care, and referrals to their collaborative care program—a difference that may be reflected in this site’s lower overall planning and implementation costs. The contrast between site 3 and site 5 highlights the potential insights that can be gathered from collecting planning and implementation costs when organizations partner to implement a new program. Partnership work faces its own challenges and may depend on synergy among the partnering organizations at the beginning of the implementation and throughout [27], but also on ongoing commitment from leadership at each organization that can help build that synergy. Tracking costs of implementation associated with successful partnerships could highlight areas of focus for potentially less successful partnerships and perhaps reduce some inefficiencies associated with faltering partnerships.

Other studies could learn from this approach to collecting costs and utilize similar spreadsheets to study implementation and sustainability of evidence-based practices, either with or without community partners. Administrators are interested in the planning and implementation costs they may face in planning for practice change. Some speculate that without these costs, administrators may overestimate costs [28]. Clinic leaders weigh costs and benefits but may focus more heavily on costs when making decisions [29]. Based on prior experiences implementing, administrators also may feel burnout related to implementing evidence-based practices if previous costs to implement were higher than expected (personal communication with Lisa Saldana on June 22, 2018). The benefit of the spreadsheet approach that we utilized was our ability to both (1) gather information retrospectively on planning + implementation costs and (2) present a simple data collection tool that could gather prospective cost of care data from site contacts with regular support from our study team. One promising web-based tool known as the Cost of Implementing New Strategies (COINS) offers support for prospectively collecting planning + implementation costs across all stages of implementation outlined in the Stages of Implementation Completion (SIC) [30]. The SIC is a tool that can support implementation more generally or be tailored to specific implementations like collaborative care. Prospective data collection is ideal to avoid some recall bias and loss of information due to turnover of key staff or ending of formal partnerships. However, COINS data must be collected with the SIC, adding to the level of data collection overall, but offering SIC data that describes implementation progress. For most sites, we were able to successfully collect planning + implementation cost data given (1) limited lag time between the grant award date and data collection and (2) appropriate guidance documents and technical assistance support. As these data collection activities were not specified in the original grant agreements for sites, we included substantial honoraria to facilitate data collection and compensate for time spent on these activities—a total of $500 for each organization.

The spreadsheet approach is also a simple way to collect cost of care data during the sustainment period that can be tailored to an evidence-based practice, including any unique adaptations such as adding community partners to the program. We collected this data at one time point between 2 and 9 months following the end of the sites’ implementation periods. Cost of care spreadsheets could be collected repeatedly during the sustainment period so sites can maintain fidelity to the evidence-based program but address any inefficiencies to improve sustainability. Additional data collection beyond the cost of care spreadsheets may also be helpful to capture costs of sustainability that may not arise in these 1-month snapshots such as training new staff following turnover and training boosters to promote fidelity during the sustainment period. Collecting these additional training costs during the sustainment period is an important avenue for future research.

Limitations included the retrospective nature of data collection for planning + implementation costs and turnover within sites, including turnover among staff and leadership holding key information on planning + implementation activities. Two sites also faced challenges within their partnerships that resulted in them dissolving their formal partnerships. Despite these limitations, with moderate technical assistance support and a reasonable honorarium, we were able to collect complete planning + implementation data on more than half of the sites and complete data on several of the other organizations even when their partner’s data could not be completed. We delayed data collection on costs of care for sites that needed more time (e.g., to revise workflows following the end of a formal partnership for this program) and thus have more complete data on costs of care at the sites. Costs of care data was more complete due to the prospective nature of data collection, shorter time to complete this spreadsheet, and our emphasis that this information is more immediately relevant to the sites when planning for sustainability. We also split the $500 honorarium such that sites were further incentivized to complete the second spreadsheet on cost of care data.

Partnering with community agencies and to some degree family members in care is the direction health care is heading at a national level, yet planning and implementation costs associated with such partnerships are seldom studied or reported. Clinics are increasingly collecting health-related social and behavioral measures in EHRs, which is partially motivated by existing linkages between clinics and community-based resources. These measures motivate deeper connections with social service agencies as was evidenced in the development of several of the enhanced collaborative care programs in this implementation project. Such partnership work is aligned with the work happening within many Accountable Care Organizations and developing Accountable Health Communities. California and Washington State for example have developed Accountable Communities of Health that focus on community organizations that can serve as a bridge to build an infrastructure between clinics and CBOs. Partnership work will require time and regular meetings to develop these relationships and plan future directions and methods to evaluate their work together, time that is often not accounted for in the implementation of evidence-based interventions.

## Conclusion

Implementing and sustaining partnered innovations requires substantial financial resources and time, resources that are often not studied given that information on planning and implementation costs in more traditional implementations are often not collected. Such partnership work can improve services for older adults and patients with complex health and social service needs. While many factors will affect a partnership’s potential for long-term success, indicators of potential challenges may be reflected in each organization’s planning and implementation costs (e.g., limited leadership involvement). Such cost information can thus help future partnerships implementing evidence-based programs predict costs for certain activities and plan accordingly. Cost of care data is particularly helpful for sites in planning to sustain their newly implemented programs. Assessing such costs in future studies is feasible and can inform future partnership efforts to implement evidence-based programs within Accountable Care Organizations and Accountable Health Communities.

## Additional file

Additional file 1: Cost categories in Excel data collection spreadsheets. (DOCX 25 kb)

## Abbreviations

CBO: Community-based organizations
CHWs: Community health workers
EHR: Electronic health record
IT: Information technology
LCSW: Licensed clinical social worker
PCC: Primary care clinic
PCP: Primary care provider
QI: Quality improvement
VA: Veterans Administration
