# Supplementary material for: Costs of implementing and sustaining enhanced collaborative care programs involving community partners
Source: Implement Sci. 2019 Apr 18;14:37. doi: 10.1186/s13012-019-0882-6 (PMC6471861; doi:10.1186/s13012-019-0882-6)
Supplement: Supplementary file 1 — Cost categories in Excel data collection spreadsheets. (DOCX 25 kb) [file 13012_2019_882_MOESM1_ESM.docx]

**Additional File 1 – Cost Categories in Excel Data Collection Spreadsheets**

***Cost of Planning and Implementation Activities***

| **Activity** | **Staff Name** | **Title/Position** | **Salary or Hourly Wage** | **Time Spent**  **(15 min increments)** | **Additional Non-personnel Costs** |
| --- | --- | --- | --- | --- | --- |
| Developing New Roles | | | |  |  |
|  |  |  |  |  |  |
|  |  |  |  |  |  |
|  |  |  |  |  |  |
| Care Delivery and Infrastructure Development (e.g., workflow development) | | | |  |  |
|  |  |  |  |  |  |
|  |  |  |  |  |  |
|  |  |  |  |  |  |
| Strategic Decision Making | | | |  |  |
|  |  |  |  |  |  |
|  |  |  |  |  |  |
|  |  |  |  |  |  |
| IT System Development | | | |  |  |
|  |  |  |  |  |  |
|  |  |  |  |  |  |
|  |  |  |  |  |  |
| Other Costs | | | |  |  |
|  |  |  |  |  |  |
|  |  |  |  |  |  |

***Cost of Care Activities***

| **Activity** | **Staff Name** | **Title/Position** | **Salary or Hourly Wage** | **Time Spent**  **(15 min increments)** | **Additional Non-personnel Costs** |
| --- | --- | --- | --- | --- | --- |
| Care Manager Activities | | | |  |  |
|  |  |  |  |  |  |
|  |  |  |  |  |  |
|  |  |  |  |  |  |
| Psychiatric Consultant and PCP Champion Activities | | | |  |  |
|  |  |  |  |  |  |
|  |  |  |  |  |  |
|  |  |  |  |  |  |
| Other Staff Activities | | | |  |  |
|  |  |  |  |  |  |
|  |  |  |  |  |  |
